# Digital Tools for Mild Cognitive Impairment: A Systematic Review and Meta-analysis of Diagnostic Accuracy and Methodological Challenges

**DOI:** 10.1007/s11065-025-09680-8

**Published:** 2025-10-22

**Authors:** Aurora Bonvino, Ester Cornacchia, Giorgia Francesca Scaramuzzi, Daphne Gasparre, Valerio Manippa, Davide Rivolta, Paolo Taurisano

**Affiliations:** 1 Dipartimento di Biomedicina Traslazionale e Neuroscienze – DiBraiN, Università Degli Studi Di Bari, Bari, Italy; 2 Dipartimento di Medicina di Precisione e Rigenerativa e Area Jonica – DiMePRe-J, Università Degli Studi Di Bari, Bari, Italy; 3 Dipartimento di Scienze Della Formazione, Psicologia e Comunicazione – For.Psi.Com, Università Degli Studi Di Bari, Bari, Italy

**Keywords:** MCI, Early diagnosis, Digital assessment, Reliability, Cognitive decline, Neurocognitive disorders

## Abstract

Mild cognitive impairment (MCI) represents an intermediate stage between typical aging and early cognitive decline. As such, an early and accurate diagnosis is essential in making timely interventions. Digital tools, including mobile applications, web platforms, wearable devices, and artificial intelligence-driven systems, have been developed and validated to capture multidimensional data, offering innovative screening solutions. This meta-analysis aims to evaluate the diagnostic accuracy of digital tools for MCI detection in different populations and settings, with a particular focus on three key issues: (i) the overall diagnostic performance of digital tools, (ii) the influence of methodological quality of studies, and (iii) the impact of demographic factors and familiarity with technologies on diagnostic accuracy. This meta-analysis assessed diagnostic accuracy across 32 studies, reporting pooled sensitivity (0.808, 95% CI: 0.775–0.838) and specificity (0.795, 95% CI: 0.757–0.828), but with considerable heterogeneity (*I*^2^ = 71.5% sensitivity; 84.0% specificity). The HSROC analysis revealed significant intrinsic variability (*τ*ₐ = 0.807) and minimal threshold variability (*τθ* = 0.291). Meta-regression indicated that applicability concerns significantly reduced specificity (*p* = 0.037), with older age also predicting lower specificity (*p* = 0.029). Thus, implementing standardized protocols, rigorous validation processes, and targeted adaptations are crucial steps for enhancing the effectiveness of digital tools in detecting MCI.

**Supplementary Information:**

The online version contains supplementary material available at https://doi.org/10.1007/s11065-025-09680-8.

## Introduction

Mild cognitive impairment (MCI) is considered a critical stage between normal age-related cognitive changes and the early stages of neurodegenerative disorders, such as Alzheimer's disease. This condition is clinically relevant because individuals with MCI face an increased risk of progressing to dementia, with conversion rates up to 10–15% per year (Farias et al., 2009; Petersen et al., 1999). It is characterized by an impairment in one or more cognitive domains that do not interfere with individuals’ daily activities and autonomy (Petersen, 2009; Petersen et al., 1999). Early detection of MCI allows timely interventions to delay or prevent progression to dementia, preserving quality of life and alleviating the growing societal and economic burden associated with neurocognitive disorders (Garcia, 2023; Lee, 2023). However, diagnosing MCI remains challenging due to the nuanced differentiation with age-related cognitive decline (Petersen et al., 1999; Salthouse, 2010).

In this context, digital tools, including mobile applications, web-based platforms, wearable devices, and AI-driven algorithms (Sabbagh et al. 2020) , have emerged as innovative and potentially transformative methods for MCI detection. Compared to traditional cognitive assessments, digital tools offer several distinct advantages. They allow for the collection of diverse and multidimensional data, including physiological markers, phonological and linguistic characteristics, eye-tracking measures, and behavioral patterns (Harris et al., 2024; Magno et al., 2024), capturing subtle changes often overlooked in standard clinical settings. Furthermore, remote assessment allows neuropsychological evaluations for those living in rural or underserved areas (Zammit et al., 2021), hence democratizing early detection and broadening the reach of cognitive health intervention.

Magno et al. (2024) conducted a systematic review and meta-analysis to evaluate the diagnostic accuracy of digital solutions for cognitive impairment screening, analyzing data from 25 studies. They categorized the digital solutions into two main groups: innovative digital solutions specifically designed for digital administration and paper-based digital solutions (digital adaptations of traditional cognitive tests). Their findings indicated variability in diagnostic accuracy: innovative digital solutions showed relatively high sensitivity but lower specificity, whereas paper-based digital solutions exhibited higher specificity but lower sensitivity. Nevertheless, demographic and contextual factors can influence diagnostic accuracy. Cognitive performance is shaped by a complex interplay of variables, such as age, education, cultural background, and technological proficiency (Hall et al., 2023; Kai, 2023; Qi & Derakhshan, 2024). These factors can affect test outcomes and interact with the design and usability of digital tools, potentially introducing bias or limiting generalizability. For instance, older adults with limited technology experience may face unique challenges when engaging with digital assessments, leading to misclassification or frustration. Addressing these challenges requires careful consideration of tool design and the implementation of adaptive testing protocols that account for individual differences in cognitive and technological capabilities.

Accordingly, Magno et al. (2024) emphasized the need for further research to confirm these promising results and recommended a thorough assessment of methodological quality before clinical implementation. Findings encourage researching whether digital tools’ characteristics, socio-demographic variables, and varying levels of internal and external validity (risk of bias vs. applicability concerns) affect diagnostic outcomes. This could help ensure a comprehensive understanding of current tool performance and guide future developments and optimizations in digital cognitive screening approaches.

Hence, this meta-analysis aims to quantify the diagnostic accuracy of digital tools, focusing on their sensitivity (ability to identify true positives) and specificity (ability to identify true negatives). High sensitivity ensures that individuals with MCI are correctly identified, enabling timely interventions, while high specificity minimizes false positives, which can lead to unnecessary stress, interventions, and healthcare costs (Garcia, 2023; Lee, 2023). In addition, this work investigates how diagnostic performance varies based on the methodological quality of the studies, particularly concerning the QUADAS-2 framework. A further objective is to examine how demographic variables, such as age, education, technological familiarity, and tool design, influence diagnostic accuracy. By addressing these critical issues, this meta-analysis poses three central research questions aiming to provide a comprehensive overview of the reliability of these tools and their potential for broad implementation, identify the methodological standards most conducive to producing reliable and generalizable results, and address the practical considerations needed to optimize digital tools for diverse populations and real-world conditions. The questions are as follows:

- **RQ1:** What is the overall diagnostic accuracy—measured in terms of sensitivity and specificity—of MCI digital screening across diverse populations and settings?
- **RQ2:** How does diagnostic performance vary based on the methodological quality of studies, as assessed by the QUADAS-2 framework?
- **RQ3:** How do demographic variables, such as age, education, tool characteristics, and administration mode influence diagnostic accuracy?

By integrating insights from methodological, demographic, and technological perspectives, this meta-analysis aims to develop more effective and equitable approaches to early diagnosis, empowering clinicians and researchers to face the growing challenge of dementia with precision and foresight.

## Materials and Method

The current review was conducted according to Preferred Reporting Items for Systematic Reviews and Meta-Analyses (PRISMA) guidelines. The protocol was registered in PROSPERO on the 6th of August 2024 (CRD42024577162).

### Search Strategy

Studies were sought using four electronic databases (Scopus, Web of Science, MEDLINE, and APA PsycINFO). Searches (last updated 30th October 2024) were prepared to include all the articles that have their titles, abstract, or keywords conform with the following Boolean expression: (“serious game” OR “applied game” OR “gamification” OR “health game” OR “mobile App” OR “smartphone app” OR “digital tool” OR “e-health” OR “digital device” OR “wearable device”) AND (“cognitive function assessment” OR “cognitive assessment” OR “cognitive screening” OR “cognitive function screening” “game-based cognitive assessment” OR “game-based cognitive screening” OR “cognitive disorder” OR “cognitive impairment” OR “cognitive decline” OR “cognitive disorder assessment” OR “cognitive impairment assessment” OR “cognitive decline assessment” OR “cognitive disorder screening” OR “cognitive impairment screening” OR “cognitive decline screening” OR “early identification of cognitive decline”). This meta-analysis was guided by the PICO framework (Population, Intervention, Comparison, Outcome):

- Population (P): Studies involving adults (> 18 years) at risk of cognitive decline were included, with a particular focus on individuals with Mild Cognitive Impairment (MCI). Participants were required to have no diagnosis of major neurocognitive disorders, no acute neurological conditions, and not to be institutionalized;
- Intervention (I): The interventions of interest were digital tools for screening cognitive decline. These included mobile apps, wearable devices, online platforms, and serious games or gamification solutions. Tools exclusively used for monitoring patients already diagnosed with cognitive impairment were excluded;
- Comparison (C): Studies had to include at least one comparison between groups, such as participants with and without cognitive impairment, to evaluate the diagnostic capacity of digital tools. This comparison could involve traditional cognitive assessment methods or healthy control (HC) group;
- Outcome (O): The primary outcomes of interest were the diagnostic accuracy of digital tools, with a particular focus on sensitivity and specificity.

### Inclusion Criteria

In accordance with the PICO framework, this meta-analysis included studies that (i) evaluated digital tools specifically designed for screening or diagnosing Mild Cognitive Impairment (MCI), explicitly excluding tools solely intended for monitoring individuals already diagnosed with MCI; (ii) included adult participants (≥ 18 years old); (iii) explicitly excluded participants diagnosed with psychiatric disorders, acute neurological conditions, major neurocognitive disorders, or individuals who were institutionalized; (iv) employed case–control, cross-sectional, or cohort study designs that enabled clear identification and comparison of two distinct groups (individuals with MCI and HC or cognitively normal individuals) and compared digital tools either to a validated traditional cognitive assessment method (reference standard) or to a HC group, meaning that the latter was not strictly mandatory when a validated reference was available; (v) clearly defined MCI based on validated clinical criteria (e.g., Petersen criteria, DSM-5), standardized cognitive tests (e.g., Montreal Cognitive Assessment (MoCA) and Mini-Mental State Examination (MMSE)), or multidisciplinary clinical diagnoses; and (vi) reported at least one measure of diagnostic accuracy (e.g., sensitivity or specificity), or provided sufficient data to calculate these metrics.

### Data Extraction (Selection and Coding)

All retrieved references were imported to Rayyan (Ouzzani et al., 2016). Duplicates were detected and removed, thus identifying the retained and screened records by applying the inclusion criteria. All identified records were blinded (i.e., the reviewers were blind to each other’s assessments) and screened for potential relevance based on the title and abstract by three independent researchers (AB, EC, GFS). Disagreements were discussed and resolved. After these initial screening steps, full texts were independently reviewed by the authors considering the specific reference to the eligibility criteria. The inter-rater agreement was Cohen’s kappa (*κ*) = 0.964. Disagreements between the reviewers were resolved by discussion. Those articles that did not meet the inclusion criteria were removed.

### Data Extraction and General Overview of Included Studies

Two investigators (AB and EC) independently extracted relevant data from each study. Extracted variables included: study identification (first author, publication year, and country), diagnostic tool name, total and subgroup sample sizes (MCI and HC), mean age for MCI and HC groups (when reported), cognitive domain assessed, type of diagnostic tool, administration mode, reference standard used for diagnosis, and measures of diagnostic accuracy (sensitivity and specificity). Digital tools were classified according to the extent of digital adaptation: 0 = digitalized traditional (standard paper-based tests directly adapted into digital format), 1 = modified traditional (standard tests significantly modified for digital use), and 2 = innovative digital (newly developed digital instruments without traditional equivalents). Administration mode was categorized based on the supervision level during assessment: 0 = remote administration, 1 = lab-based administration with instructions only, 2 = lab-based administration with structured training, and 3 = lab-based administration under direct examiner supervision. The reference standard for diagnosing MCI varied among studies, including established clinical criteria (e.g., Petersen criteria, DSM-5), validated cognitive screening test cut-off scores (e.g., MoCA, MMSE), or multidisciplinary consensus-based clinical diagnoses. Sensitivity and specificity values were extracted directly when reported. When not explicitly provided, these measures were calculated from raw diagnostic data—true positives (TP), false positives (FP), false negatives (FN), and true negatives (TN)—using the standard formulas: Sensitivity = TP/(TP + FN); Specificity = TN/(TN + FP). The inter-rater agreement for quality assessment was *κ* = 1.0.

### Quality Assessment

Two authors (AB, EC) independently assessed the quality of individual studies using the Quality Assessment of Diagnostic Accuracy Studies-2 (QUADAS-2). QUADAS-2 is a validated tool used to assess diagnostic accuracy studies (Whiting et al. 2011), clearly distinguishing between Risk of Bias (RoB), which refers to the internal validity and methodological rigor of each included study, and Applicability Concerns (AC), which indicate the external validity or generalizability of the study methods and findings to clinical practice. QUADAS-2 comprises four domains: patient selection (RoB1, AC1), index test (RoB2, AC2), reference standard (RoB3, AC3), and flow and timing (RoB4) (see Tables S1 and S2 for detailed criteria and definitions). Cohen’s Kappa for quality assessment was *κ* = 0.985. Discrepancies in the quality assessment were resolved by discussion, with GFS serving as a third reviewer. Each domain was rated as having “Low”, “High”, and “Unclear” risk of bias, and applicability concerns were also assessed for the first three domains using the same rating scale.

### Data Analysis

Stata (www.stata.com) was used to perform statistical procedures to evaluate diagnostic performance across the included studies. Diagnostic test accuracy was primarily evaluated using the Hierarchical Summary Receiver Operating Characteristic (HSROC) model implemented by the metandi command in Stata, as described by Rutter and Gatsonis (2001). This model jointly estimates logit-transformed sensitivity and specificity, explicitly accounting for their correlation as well as within- and between-study variability. The HSROC model addresses threshold effects arising from varying diagnostic criteria, providing estimates of overall diagnostic accuracy (*θ*), threshold parameters (*β*), ROC curve shape (*α*), between-study variances (*τ*^2^, derived via a custom script as metandi outputs summary variances, Σ), and correlation between sensitivity and specificity (*ρ*). Graphical visualization of the HSROC summary operating point, ROC curve, confidence regions, and prediction ellipses was produced using metandiplot. Additionally, individual study estimates of sensitivity and specificity were visually summarized using forest plots generated by the metan command, providing a descriptive overview of study-level heterogeneity. Separate random-effects univariate meta-regressions were conducted for sensitivity and specificity, each on the logit scale and weighted by the inverse of its variance. Although this approach does not account for the correlation between the two measures, it was appropriate given the analytical aim of assessing the impact of study-level covariates on each parameter independently. Univariate meta-regression is a recognised method in diagnostic accuracy research (Deeks et al., 2005; Zwinderman & Bossuyt, 2008), particularly suitable for exploring heterogeneity when sensitivity and specificity may be influenced by distinct sources of bias or design variability. Potential sources of heterogeneity were investigated via meta-regression analyses (metareg), incorporating QUADAS-2 assessments (overall Risk of Bias and domain-specific RoB1–RoB4; overall Applicability Concerns and domain-specific AC1–AC3) and study-level covariates such as mean age, diagnostic tool type, and mode of administration. Variables such as education and family history were excluded due to limited and inconsistent reporting across studies.

### Data Synthesis and Application of the Grading of Recommendations Assessment, Development, and Evaluation (GRADE) Framework

The diagnostic accuracy of digital tools for identifying MCI was assessed using the Grading of Recommendations Assessment, Development, and Evaluation (GRADE) framework (Guyatt et al., 2008a, b). GRADE evaluates the certainty of evidence across five domains: risk of bias, inconsistency, indirectness, imprecision, and publication bias.

Study limitations were systematically evaluated using the QUADAS-2 tool across Risk of Bias and Applicability Concerns: Patient Selection, Index Test, Reference Standard, and Flow and Timing. Each domain was rated as Low, Unclear, or High risk based on predefined criteria, summarizing the proportion of studies per risk category.

Inconsistency was evaluated using the *I*^2^ statistic, indicating heterogeneity across studies. Intrinsic accuracy variability (*τ*ₐ) and minimal threshold variability (*τθ*) were assessed to differentiate intrinsic diagnostic differences from threshold effects. Meta-regression analyses identified significant predictors contributing to observed heterogeneity.

Indirectness evaluated how closely studies reflected clinical populations, settings, and diagnostic digital tools in clinical practice.

Imprecision was assessed by examining the width of the confidence intervals (CI) around pooled sensitivity and specificity estimates. Wider intervals indicated greater uncertainty.

Publication bias was evaluated visually and statistically using funnel plots, considering asymmetry as indicative of potential bias. The overall GRADE certainty was categorized as High (⊕⊕⊕⊕), Moderate (⊕⊕⊕○), Low (⊕⊕○○), or Very Low (⊕○○○).

## Results

### Literature Search and Selection Process

**Fig. 1:**
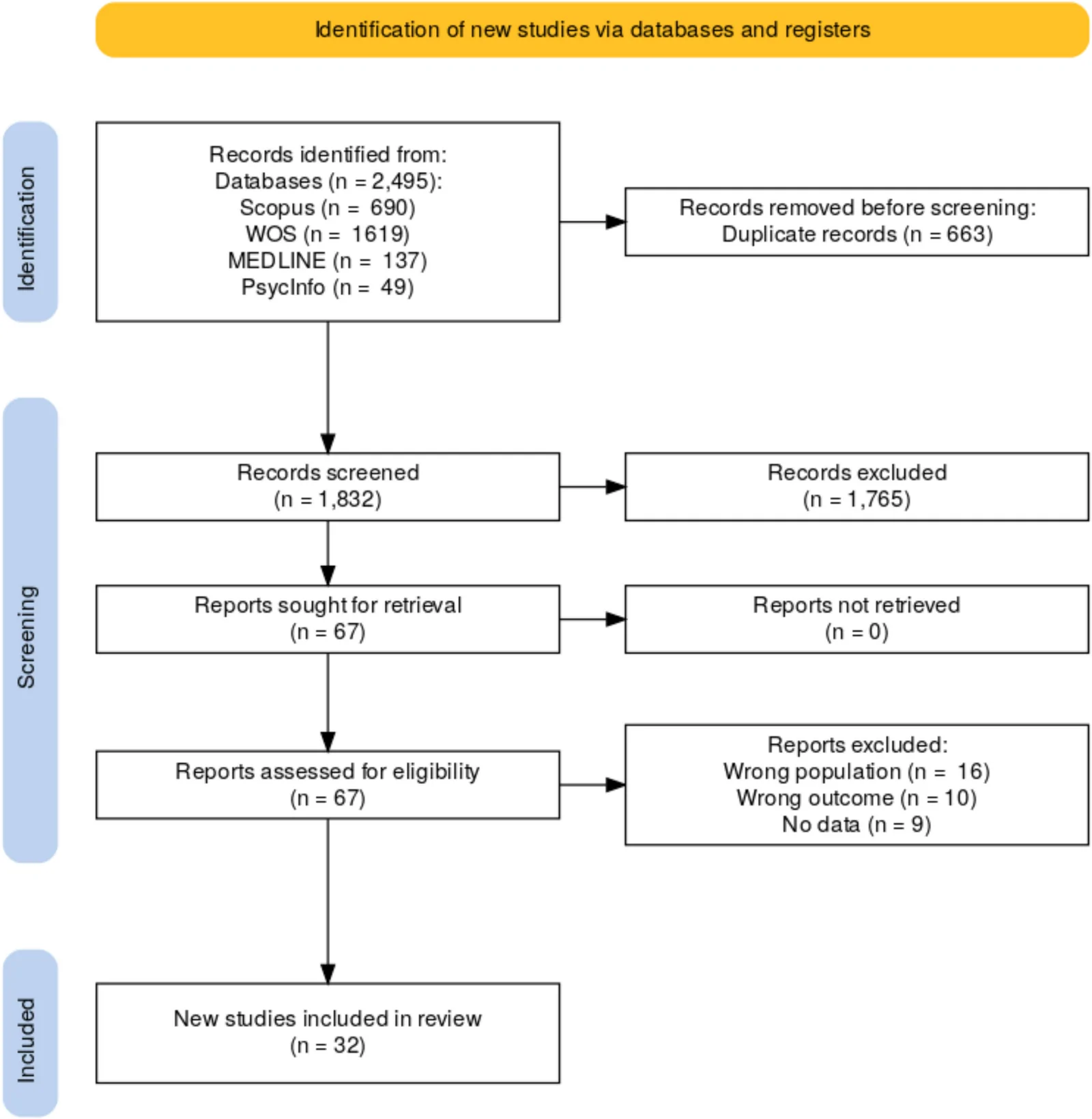
PRISMA flow diagram illustrating the study selection process

The search retrieved 2495 studies, with 663 duplicates removed. After screening titles and abstracts, 67 studies were identified for full-text review. Thirty-five were dropped by the exclusion criteria; hence, 32 articles were included. The majority of the studies were excluded due to the wrong population (i.e., studies that only considered the clinical condition and not MCI) or wrong outcomes (i.e., studies that did not report diagnostic accuracy analyses or did not consider the assessment of cognitive domains). The selection process is summarized in Fig. 1, while descriptions of included articles are reported in Table S1.

### Sample Characteristics

The 32 studies included originated from multiple countries (e.g., Spain, Italy, USA, and China). The sample sizes ranged from 50 participants (Müller et al., 2017) to 606 participants (Fung & Lam, 2020), with the MCI group typically comprising 30% to 60% of the total sample. The total number of participants across all studies was 4786, with 1932 participants in the MCI group and 2854 HC. The mean age for MCI participants was 72.64 years, and 70.54 years for HC, with an overall mean age of 71.59 years. The studies included assess various cognitive domains using both traditional and novel test types. Twenty-one studies used digitalized traditional tests, while 11 employed novel tests. The mode of administration was predominantly laboratory-based, used in 27 studies, while remote administration accounted for 5 studies. Nine studies, 8 laboratory-based and 1 remote, involved training-based administration. Four traditional single-domain tests focused on memory, 4 on visuo-spatial assessments, and one on language. Most of the multi-domain tests combined memory and language assessment (*N* = 4) or memory and attention (*N* = 4); only one study combined multi-domain assessment through memory and visuo-spatial functions, while two studies combined three domains (i.e., memory, executive functions, and attention). Novel tests predominantly evaluated, through multi-domain assessments, memory and executive function (*N* = 6), followed by memory and attention (*N* = 2). Other combinations in novel tests included attention and executive function (*N* = 2), and memory, attention, and problem-solving (*N* = 1). MCI diagnoses were primarily based on the Petersen Criteria, MoCA, and MMSE.

### QUADAS-2 Assessment

**Fig. 2:**
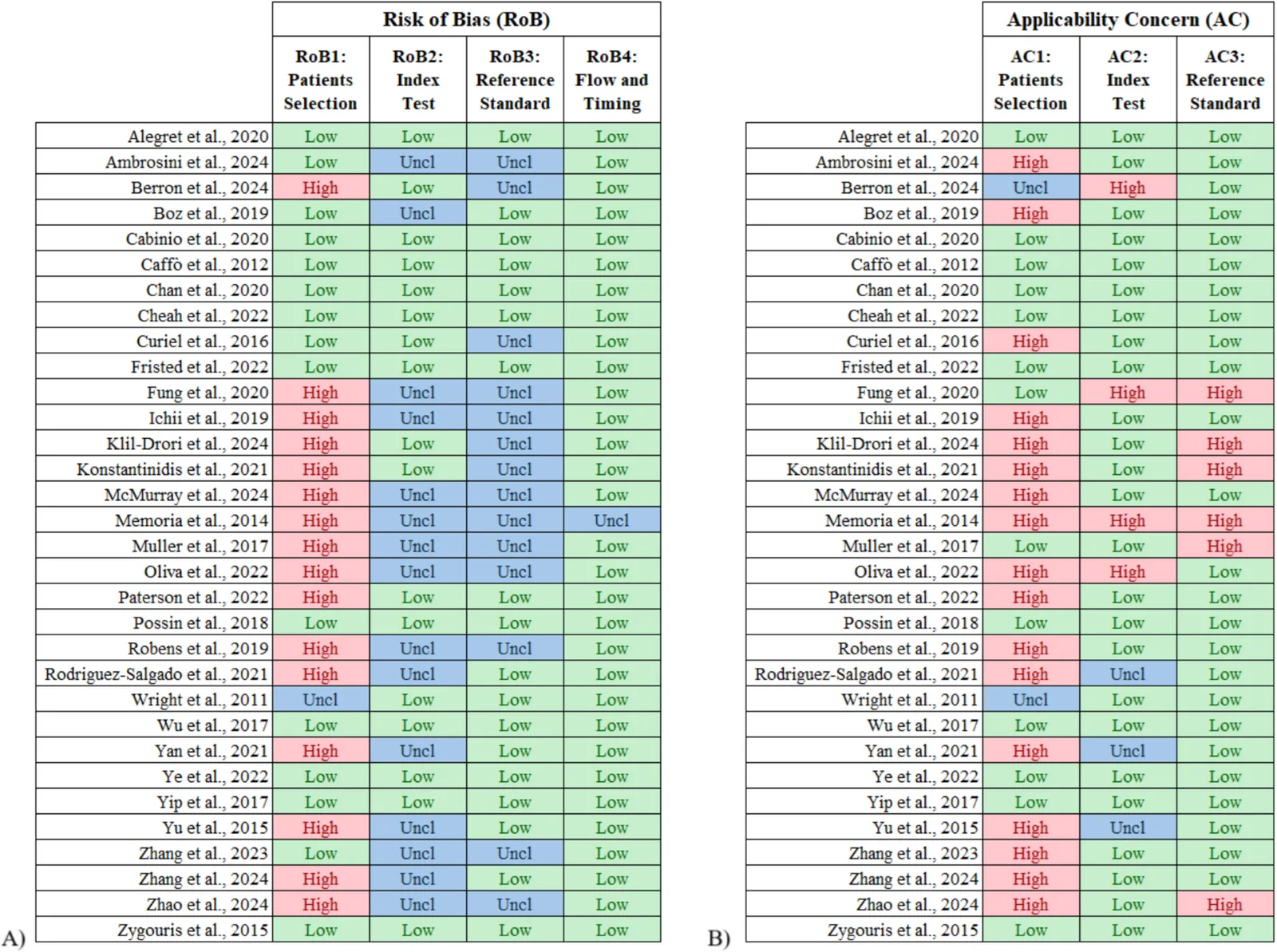
QUADAS-2 ratings for: (**A**) Risk of Bias (RoB) domains and (**B**) Applicability Concerns (AC) domains. Studies are categorized as having low (green cells), unclear (blue cells), or high (red cells) RoB or AC

The QUADAS-2 assessment of risk of bias and applicability concerns revealed distinct patterns across the studies (Fig. 2). In the assessment of Risk of Bias (RoB), the domain "Flow and Timing" had the highest percentage of low-risk studies (96.9%), suggesting strong methodological rigor regarding the timing and sequence of tests. In contrast, the domains “Patient Selection” and “Reference Standard” each showed moderate methodological reliability, with half (50%) of the studies rated as low risk. The “Index Test” domain exhibited a slightly better result, with 53.1% low-risk studies.

Regarding Applicability Concerns (AC), the domain “Index Test ” had the highest proportion of studies with low applicability concerns (87.5%), followed closely by “Reference Standard” at 81.3%. However, the “Patient Selection” domain highlighted issues with the generalizability and relevance of patient populations included in the studies (low concern = 46.9%).

### Meta-analysis Overall Results

The meta-analysis included 32 studies and evaluated the diagnostic accuracy of digital tools. Using the hierarchical summary receiver operating characteristic (HSROC) model, the overall sensitivity was estimated to be 0.808 (95% CI: 0.775–0.838), and specificity was 0.795 (95% CI: 0.757–0.828), suggesting robust performance in correctly classifying both true positive and true negative cases. Cochran’s *Q* indicated significant heterogeneity for both sensitivity (*Q* = 108.85, *p* < 0.001, *I*^2^ = 71.5%) and specificity (*Q* = 194.12, *p* < 0.001, *I*^2^ = 84.0%).

**Fig. 3:**
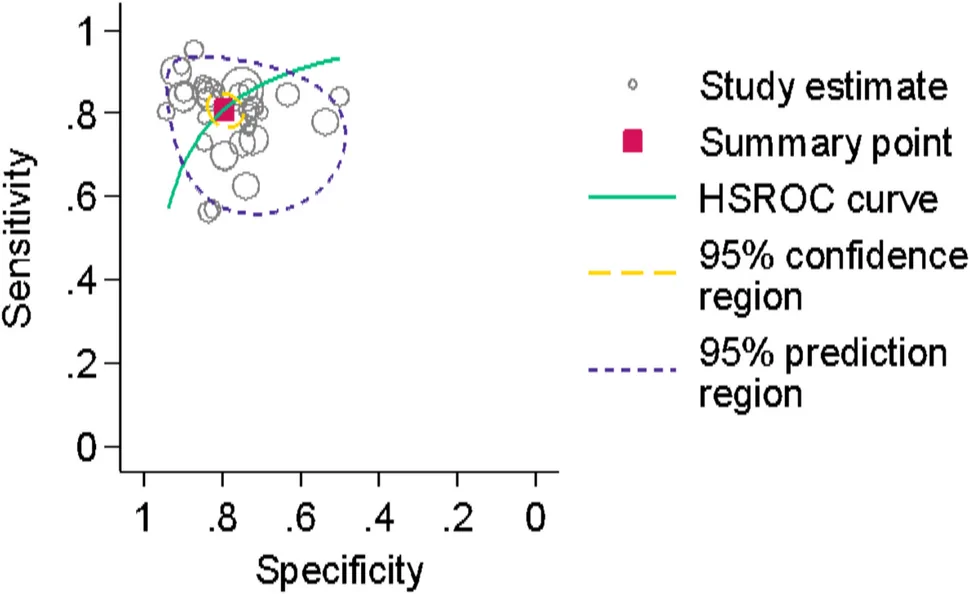
HSROC Plot of Diagnostic Test Performance. The HSROC plot illustrates the diagnostic performance of the test across the included studies. The solid line represents the summary curve for sensitivity and specificity, with the dots showing each individual study estimate. The dashed lines indicate the confidence intervals for the sensitivity and specificity values, reflecting the uncertainty in the estimates. The midpoint of the plot represents the overall diagnostic accuracy, accounting for both sensitivity and specificity

**Fig. 4:**
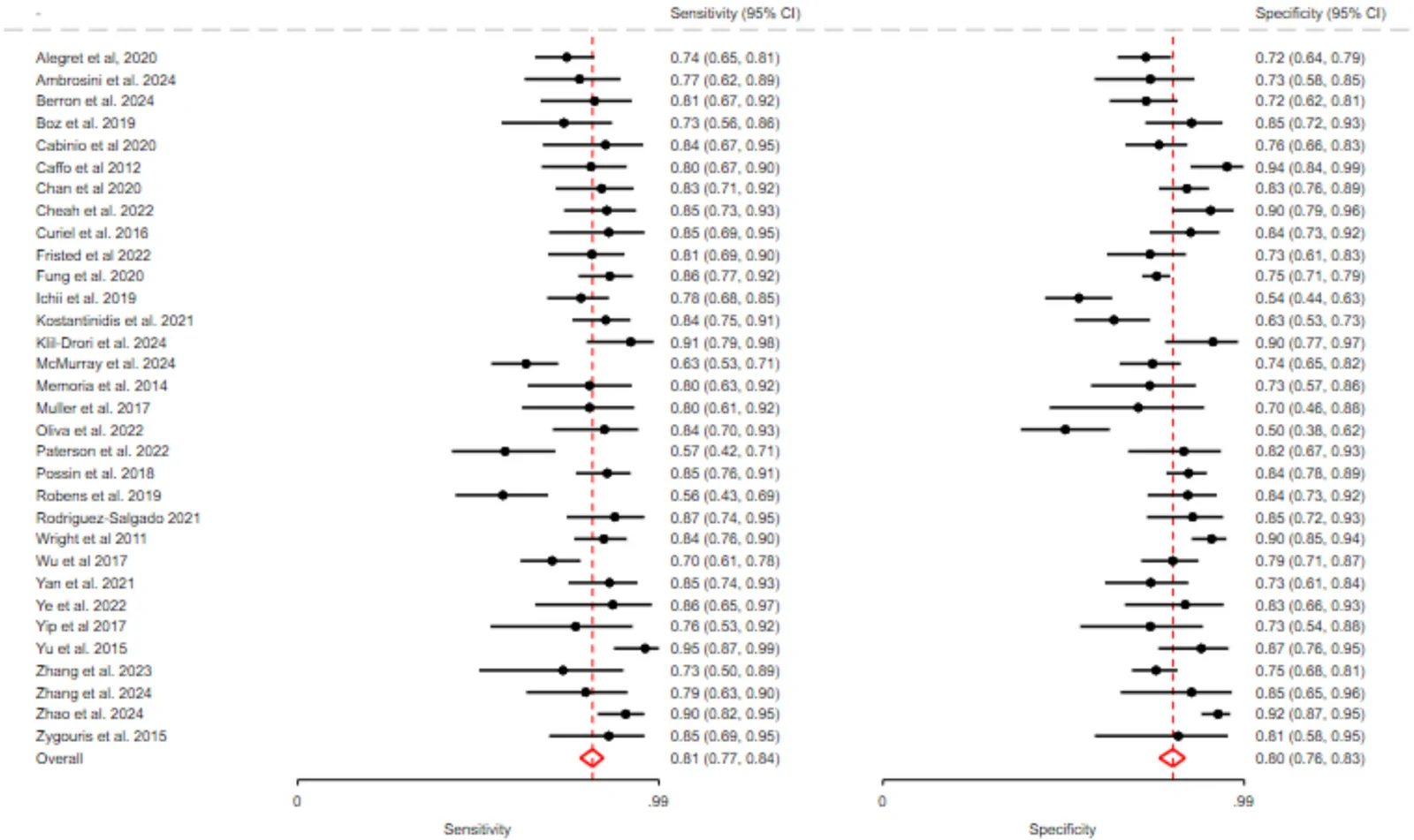
Forest plot illustrates sensitivity and specificity values of digital tools for Mild Cognitive Impairment (MCI) screening across included studies. Each horizontal line represents the 95% confidence interval (CI) for individual study estimates, while the central square indicates the point estimate. The diamond shape represents the overall pooled sensitivity and specificity estimates with corresponding 95% CI derived from random-effects meta-analysis

HSROC model parameters provided additional insights into overall diagnostic accuracy and potential threshold effects. The accuracy parameter (lambda, *λ*) was estimated at 2.809 (95% CI: 2.474–3.144), confirming strong overall diagnostic performance. The threshold parameter (theta, *θ*) was 0.153 (95% CI: −0.188 to 0.494), indicating minimal influence of threshold variations on diagnostic accuracy, and the shape parameter (beta,* β*) was 0.160 (*p* = 0.510), which was not statistically significant, reinforcing that threshold variability had a negligible impact on performance. The HSROC model further described heterogeneity on the latent scale, with accuracy-related variability *s*^2^*α* = 0.651 (95% CI: 0.338–1.255; *τ*ₐ = 0.807) and threshold-related variability *s*^2^*θ* = 0.085 (95% CI: 0.039–0.184; *τθ* = 0.291). Thus, heterogeneity between studies primarily stemmed from differences in intrinsic accuracy rather than threshold variations. Taken together, the HSROC analysis confirmed robust diagnostic accuracy, and the *τ* metrics derived from the HSROC model clarified that absolute variability between studies remained limited, despite substantial relative heterogeneity (Table 1, Fig. 3). Sensitivity and specificity forest plots are reported in Fig. 4.

**Table 1:** Overall diagnostic accuracy HSROC model parameters

Parameter	Estimate	SE	95% CI	*p*-value	*τ* (logit)
Sensitivity (Se)	0.808	0.016	0.775 to 0.838	–	0.076
Specificity (Sp)	0.795	0.018	0.757 to 0.828	–	0.089
HSROC lambda (*λ*)	2.809	0.171	2.474 to 3.144	–	–
HSROC theta (*θ*)	0.153	0.174	−0.188 to 0.494	–	–
HSROC beta (*β*)	0.160	0.242	−0.315 to 0.634	0.510	–
HSROC *s*^2^*α* (accuracy)	0.651	0.218	0.338 to 1.255	–	0.807
HSROC *s*^2^*θ* (threshold)	0.085	0.033	0.039 to 0.184	–	0.291

### Meta-regression

All the meta-regression results are reported in Table 2.

**Table 2:** Single-model analyses of digital tool characteristics predicting sensitivity and specificity. Regression coefficients (*β*), standard errors (SE), *p*-values, between-study variance (*τ*^2^), heterogeneity (*I*^2^), and adjusted *R*^2^ values are reported for each predictor, including risk of bias domains (RoB1–4, overall), applicability concerns (AC1–3, overall), mean age of HC and MCI groups, type of digital tool, and administration mode

Predictor	Outcome											
	Sensitivity						Specificity					
	*β*	SE	*p*-value	*τ*^2^	*I*^2^ (%)	Adj *R*^2^ (%)	*β*	SE	*p*-value	*τ*^2^	*I*^2^ (%)	Adj *R*^2^ (%)
RoB overall	−0.03	0.22	0.877	0.2051	67.9	−4.7	−0.19	0.23	0.429	0.2843	79.4	−1.7
RoB1	0.02	0.21	0.928	0.2059	67.9	−5.1	0.01	0.22	0.964	0.2919	80.1	−4.4
RoB2	−0.07	0.21	0.723	0.2037	67.6	−4.0	−0.27	0.22	0.225	0.2752	78.9	1.6
RoB3	−0.02	0.21	0.917	0.2055	67.9	−4.9	−0.38	0.21	0.080	0.2517	77.2	10.0
AC overall	−0.02	0.23	0.930	0.2058	67.9	−5.1	−0.29	0.25	0.258	0.2789	79.2	0.2
AC1	−0.07	0.21	0.738	0.2028	67.4	−3.5	0.10	0.22	0.639	0.2571	79.0	8.0
AC2	0.22	0.31	0.484	0.1965	66.4	−0.3	**−0.64**	**0.30**	**0.038**	**0.2452**	**78.6**	**12.3**
AC3	0.49	0.25	0.059	0.1629	61.5	16.9	0.00	0.29	1.000	0.2921	80.2	−4.5
MCI mean age	0.00	0.01	0.782	0.2552	71.5	−5.6	0.00	0.01	0.915	0.2872	79.5	−5.8
HC mean age	0.04	0.03	0.258	0.2345	68.8	3.0	**−0.07**	**0.03**	**0.025**	**0.2102**	**73.4**	**22.6**
Administration mode	−0.08	0.11	0.453	0.1966	66.8	−0.4	0.04	0.12	0.717	0.2902	80.1	−3.8
Type	−0.01	0.12	0.949	0.2056	67.9	−4.9	−0.23	0.12	0.067	0.2389	76.0	14.5

### Methodological Quality (RoB)

Meta-regression analyses examining methodological quality (RoB) indicated limited impact on sensitivity and specificity. For sensitivity and specificity, neither single RoB indicators (RoB1, RoB2, RoB3) nor aggregated RoB reached statistical significance (all *p* > 0.05).

### Applicability Concerns (AC)

Regarding applicability concerns, analyses showed no significant relationships for sensitivity (all *p* > 0.05). For specificity, AC2 was significantly associated individually (*β* = −0.64, SE = 0.30, *p* = 0.038). Thus, applicability concerns explained about 8% of the variability in specificity.

### Age

Analyses of age showed no significant predictors for sensitivity (HC mean age, MCI mean age; all *p > *0.05). However, the mean age of HC was a significant negative predictor of specificity (*β* = −0.072, SE = 0.030, *p* = 0.025). This indicates a relationship between higher control age and reduced diagnostic specificity.

### Digital Tool Characteristics and Administration Mode

For digital tool characteristics, analyses showed no significant relationships for sensitivity (all *p* > 0.05). For specificity, the type of digital tool approached significance individually (*β *= −0.23, SE = 0.12, *p* = 0.067). For administration mode, analyses similarly revealed no significant relationships for sensitivity (*β* = −0.08, SE = 0.11, *p* = 0.453) or specificity (*β* = 0.04, SE = 0.12, *p* = 0.717).

### GRADE Assessment and Meta-analysis Findings

The overall assessment using the GRADE methodology resulted in a moderate level of certainty (⊕⊕⊕○) for both sensitivity and specificity of digital tools in diagnosing MCI (Table 3). This conclusion is supported by comprehensive consideration of methodological rigor, statistical reliability, and clinical applicability. A notable proportion of the included studies presented moderate methodological limitations, especially related to biases in patient selection and the administration of the index tests. These limitations introduced a moderate reduction in confidence regarding the robustness and generalizability of the results. The observed inconsistency in the diagnostic accuracy estimates was characterized by moderate heterogeneity for sensitivity (*I*^2^ = 71.5%) and pronounced heterogeneity for specificity (*I*^2^ = 84.0%), indicating substantial variability across the studies included in the analysis. This notable variability primarily stemmed from intrinsic differences in diagnostic performance among the diverse digital tools evaluated, as demonstrated by a high intrinsic accuracy variability (*τ*ₐ = 0.807). Further exploration via meta-regression analyses revealed additional contributing factors, notably concerns regarding the applicability of the index test methodologies (AC2, *p* = 0.038), marginal influences from potential biases associated with the reference standard assessments (RoB3, *p* = 0.057), and significant variability linked to the age of participants (*p* = 0.025). Collectively, these factors significantly elucidate the heterogeneity observed, particularly impacting specificity outcomes and emphasizing the need for cautious interpretation when applying these digital tools clinically.

**Table 3:** GRADE classification evidence quality

Criterion	Sensitivity	Specificity
Estimate (95% CI)	0.81 (0.77–0.84)	0.79 (0.75–0.83)
Study limitations	Moderate	Moderate
Inconsistency	Moderate	High
Indirectness	None	None
Imprecision	Moderate	Moderate
Publication bias	None	None
Overall GRADE	⊕⊕⊕○ Moderate	⊕⊕⊕○ Moderate

There was no concern regarding indirectness, as the reviewed studies directly and adequately mirrored clinical populations, settings, and procedures encountered in clinical practice. This direct alignment ensures high clinical relevance and applicability.

Moderate imprecision was evident through confidence intervals for both sensitivity (95% CI: 0.77–0.84; width = 7%) and specificity (95% CI: 0.75–0.83; width = 8%). These intervals fall within the moderate range of uncertainty (5–10%), warranting a moderate downgrade of certainty, though not severe enough to justify a more substantial downgrade. Finally, funnel plot analyses suggested negligible publication bias, with no significant evidence of asymmetry or selective reporting (Figure S1).

Collectively, the systematic and comprehensive assessment through GRADE clearly illustrates that, despite certain methodological and statistical limitations, the pooled diagnostic accuracy estimates for digital tools remain robust and clinically informative, maintaining moderate confidence (⊕⊕⊕○) in their validity and applicability for detecting MCI.

## Discussion

This meta-analysis examined the diagnostic accuracy of digital tools for detecting MCI, focusing on three primary research questions: (i) the overall diagnostic accuracy (RQ1), (ii) the impact of methodological quality on diagnostic performance as assessed by the QUADAS-2 framework (RQ2), and (iii) the influence of demographic variables and tool characteristics on diagnostic accuracy (RQ3). The analysis of 32 studies resulted in a high pooled sensitivity and specificity, but with considerable heterogeneity among them. The HSROC analysis revealed significant intrinsic variability and minimal threshold variability. Furthermore, applicability concerns and older age negatively affected specificity.

Addressing RQ1, the pooled sensitivity of 0.808 (95% CI: 0.775–0.838) and specificity of 0.795 (95% CI: 0.757–0.828) across 32 studies demonstrate robust performance, confirming the capability of digital tools to correctly classify both true positive and negative cases. The significant heterogeneity identified through *I*^2^ statistics (sensitivity *I*^2^ = 71.5%; specificity *I*^2^ = 84.0%) indicates substantial relative variability among study results. Such high relative heterogeneity suggests that although digital tools perform well overall, individual studies vary considerably, potentially due to differences in study design, demographic characteristics, digital tool specifications, and administration procedures.

The HSROC model analysis further clarified the nature of this heterogeneity. High intrinsic accuracy-related variability (*τ*ₐ = 0.807) reveals substantial absolute differences in intrinsic diagnostic accuracy across the tools, underscoring that variability predominantly stems from genuine performance differences rather than from variations in diagnostic thresholds or test administration. Conversely, the low threshold-related variability (*τθ* = 0.291) indicates that absolute differences due to varying diagnostic thresholds among studies have limited practical impact on the observed variability. Moreover, the HSROC parameters support robust diagnostic performance, with a high accuracy parameter (*λ* = 2.809; 95% CI: 2.474–3.144) and non-significant threshold (*θ* = 0.153) and shape parameters (*β* = 0.160; *p* = 0.510), reinforcing minimal threshold influence on the heterogeneity observed.

Meta-regression analyses examining methodological quality (RoB) indicated a limited impact on both sensitivity and specificity, suggesting that variations in methodological rigor across studies did not significantly influence diagnostic performance outcomes. The absence of significant findings for (RoB1, RoB2, RoB3) and aggregated RoB (all *p* > 0.05) implies that, although methodological biases are present, their effect on diagnostic accuracy may be less pronounced or overshadowed by other factors. Regarding AC, no significant relationships emerged for sensitivity, highlighting robustness in identifying true positive cases regardless of variations in study applicability. Conversely, specificity was significantly influenced by the AC2 indicator (*β* = −0.64, SE = 0.30, *p* = 0.038). This critical finding indicates that concerns about the relevance and suitability of digital tools for the studied populations substantially impact their ability to correctly identify true negatives, thus increasing the rate of false positives. Specifically, AC2 accounted for approximately 8% of the variability in specificity, underscoring the importance of carefully aligning digital tools’ design and implementation with the specific characteristics of the clinical populations targeted. Overall, the GRADE assessment provided moderate certainty (⊕⊕⊕○) for both sensitivity and specificity, primarily influenced by moderate methodological limitations, pronounced heterogeneity, and moderate imprecision. However, the direct clinical relevance of the included studies was high, ensuring strong applicability to real-world clinical settings.

Focusing specifically on demographic factors, the increased mean age of the HC group emerged as a significant negative predictor of specificity (*p *= 0.025). This result suggests that as HC participants’ age increases, the likelihood of false positives rises, possibly because normal cognitive aging processes can resemble the mild cognitive deficits characteristic of early MCI. This finding aligns with previous studies suggesting that age-related changes in cognitive function may influence test results (Ambrosini et al., 2024; Memória et al., 2014). As individuals age, they may exhibit cognitive patterns that resemble MCI, potentially leading to a higher rate of false positives when using diagnostic tools that are not adjusted for age-related cognitive decline. However, no significant relationship was found between age and sensitivity, suggesting that digital tools remain effective in identifying MCI across different age groups. This may be due to the ability of these tools to accurately detect cognitive impairment, regardless of age, although further research is needed to assess how other demographic factors, such as education and cognitive reserve, can affect diagnostic accuracy (Zhao et al., 2024). These findings underscore the importance of considering demographic factors, such as age and technological familiarity, in the development and application of digital tools. Older individuals, particularly those with less technological experience, may face challenges in effectively using digital tools. Thus, digital MCI screening tools should prioritize user-friendliness and adaptability to accommodate diverse age groups and varying levels of technological literacy.

Additionally, digital tool type, categorized from digitalized traditional tests (minimal innovation) to fully innovative designs, showed a marginal impact on specificity individually (*p* = 0.067), suggesting that digitalized traditional tests may offer superior specificity compared to more innovative designs, possibly due to their established validity and familiarity for older populations, reducing the risk of false positives. In contrast, no significant associations were found for administration mode (i.e., remote, lab-based with instructions, lab-based with structured training, or lab-based with direct examiner supervision), with neither sensitivity (*p* = 0.453) nor specificity (*p* = 0.717) showing notable effects. These findings indicate that how the tool is administered may not significantly influence diagnostic accuracy, at least within the parameters of the current dataset.

Rather than replacing standard instruments like MoCA or MMSE, digital tools can serve as automated, brief, and self-administered preliminary screening even in remote clinical settings. Whether cognitive impairment is detected, a comprehensive neuropsychological evaluation should be scheduled, whereas null results could reduce unnecessary referrals and clinician workload. In primary care and telemedicine contexts, where resources are limited, tools like Remote Digital Memory Composite identify patients requiring further assessment (Berron et al., 2024). Effective clinical implementation requires a user-friendly design tailored for older adults, validation against established tests, clearly defined diagnostic thresholds, and integration with electronic health records. Additionally, addressing digital literacy barriers and ensuring data privacy are crucial for achieving equitable and broad adoption (Cho et al., 2024). Given the novelty of these digital tools, their practical implementation remains an evolving area. Digital cognitive screening methods are likely most beneficial in primary care and community settings, serving as preliminary, accessible assessments that efficiently identify individuals for further evaluation. Optimal thresholds for real-world acceptance typically emphasize sensitivity above 0.80 to minimize missed diagnoses, coupled with specificity above 0.70 to reduce false positives. Therefore, integrating digital tools into clinical workflows should involve careful consideration of these thresholds, clear referral pathways, and ongoing validation studies to ensure their accuracy and reliability in diverse populations. Standardized protocols would ensure that digital tools are assessed under comparable conditions, leading to more reliable and generalizable results (Berron et al., 2024; Paterson et al., 2022). Implementing standardized procedures should encompass all aspects of the research, from participant selection and test administration to data analysis. Additionally, ensuring that digital tools undergo rigorous validation against established diagnostic standards will increase their credibility and acceptance in clinical practice (Cabinio et al., 2020). These efforts will be critical for maximizing the utility of digital tools in MCI screening and ensuring that they are effective across diverse patient populations.

## Conclusions, Limitations, and Future Perspectives

This meta-analysis confirms the effectiveness of digital tools for detecting MCI, highlighting their high sensitivity and specificity. The high sensitivity observed suggests that digital tools effectively identify individuals who may benefit from early intervention (Alegret et al., 2020), able to slow the progression of cognitive decline and delay dementia onset (Yu et al., 2015). This shows that digital screening methods can accurately identify individuals with cognitive impairment and those without, making them highly promising for clinical applications.

Nevertheless, the analysis uncovered significant heterogeneity among studies, primarily due to intrinsic differences in performance among the digital tools examined, rather than methodological biases or variations in diagnostic thresholds, emphasizing the necessity of careful tool selection and validation. Specificity, in particular, is significantly affected by clinical applicability concerns and demographic factors, notably the advanced age of HC participants. The significant negative relationship between age and specificity underscores the importance of tailoring digital tools to the needs of older adults. Age-related cognitive changes can pose challenges for older populations using digital tools, ensuring a design that minimizes false positives, which is critical for their widespread adoption in clinical settings (Fung & Lam, 2020; Oliva & Losa, 2022).

Furthermore, it is important to acknowledge that other potentially influential moderators, such as educational level and tech literacy, could not be analyzed due to insufficient or inconsistent reporting across included studies. Future research should aim to systematically evaluate these factors to better understand their impact on the effectiveness and accessibility of digital tools for MCI screening.

In conclusion, digital tools demonstrate substantial promise for the early detection of MCI and the facilitation of timely intervention strategies. However, the adoption of standardized protocols, comprehensive validation procedures, and careful customization to demographic characteristics are essential steps to ensure their robustness, accuracy, and generalizability in clinical practice.

## Supplementary Information

Below is the link to the electronic supplementary material.

ESM1 (DOCX.378 KB)

## Data Availability

The data that support the findings of this study are available on request to the first author (AB).
