# Supplementary material for: Digital Tools for Mild Cognitive Impairment: A Systematic Review and Meta-analysis of Diagnostic Accuracy and Methodological Challenges
Source: Neuropsychol Rev. 2025 Oct 22;36(3):375–87. doi: 10.1007/s11065-025-09680-8 (PMC13653904; doi:10.1007/s11065-025-09680-8)
Supplement: Supplementary file 1 [file 11065_2025_9680_MOESM1_ESM.docx]

**Table S1. QUADAS-2 risk-of-bias domains and judgement criteria**

Definitions of the four QUADAS-2 domains (Patient Selection, Index Test, Reference Standard, Flow & Timing) and the signalling questions that underpin each judgement. For every domain, the table details the conditions that lead to a low, high, or unclear risk-of-bias rating, enabling transparent appraisal of the internal validity of the included diagnostic-accuracy studies.

| **QUADAS-2 Domains** | **Key signalling questions** | **Low risk** | **High risk** | **Unclear** |
| --- | --- | --- | --- | --- |
| **RoB1 Patient Selection** | Were participants consecutively or randomly selected? | Representative sample obtained by consecutive or random sampling, no unjustified exclusions. | Convenience sample, case-control study design, or inappropriate exclusions. | Insufficient detail on sampling methods. |
|  | Was case-control design avoided? |  |  |  |
|  | Were exclusions appropriate? |  |  |  |
| **RoB2 Index Test** | Was the test interpreted blind to the reference standard? | Test interpretation was blinded to diagnosis, threshold was pre-specified independently. | Test interpretation was not blinded, threshold was data-driven or chosen after results seen. | Blinding or threshold specification unclear. |
|  | Was the diagnostic threshold pre-specified? |  |  |  |
| **RoB3 Reference Standard** | Does the reference correctly classify the condition? | Reliable “gold standard” independently interpreted, clearly classifies disease status. | Imperfect reference standard, interpretation influenced by index test results. | Validity or independence of reference unclear or poorly described. |
|  | Was interpretation independent of the index test? |  |  |  |
| **RoB4 Flow and Timing** | Was the interval between index test and reference appropriate? | Short interval between tests, consistent reference for all, complete follow-up. | Long or variable intervals, different references used, patients excluded after testing. | Insufficient information about timing, consistency, or completeness. |
|  | Was the same reference standard used for all patients? |  |  |  |
|  | Were all patients analyzed? |  |  |  |

**Table S2. QUADAS-2 applicability-concern domains and judgement criteria**

Descriptions of the three applicability domains (Patient Selection, Index Test, Reference Standard) together with the key features evaluated and the thresholds for low, high, or unclear concern. The table guides readers in determining how well each study population, test procedure, and reference standard align with the review’s clinical question, and therefore how generalisable the accuracy estimates are to routine practice.

| **Domain** | **Focus of Concern** | **Low Concern** | **High Concern** | **Unclear** |
| --- | --- | --- | --- | --- |
| **AC1 Patient Selection** | Representativeness of study participants compared to the target clinical population | Study includes patients similar to those seen in routine clinical settings. | Study uses highly selective population, e.g., volunteers, hospital inpatients only. | Description of population is too vague to judge. |
| **AC2 Index Test** | Whether the execution and interpretation of the index test match routine clinical practice | Test procedure, threshold, and reader expertise reflect usual use in practice. | Test used with experimental software, altered scoring, or interpreted by researchers only. | Details on test administration, threshold, or interpretation not provided. |
| **AC3 Reference Standard** | Appropriateness of the reference standard used to define the target condition | Reference standard aligns with accepted diagnostic criteria used in current practice. | Outdated criteria, unclear clinical definitions, or proxy measures. | Diagnostic criteria or procedures not sufficiently described. |

**Table S3.** Overview of the studies included in the meta-analysis

| **Study & Tool Name (Author, Year, Country)** | **Sample Size (MCI, HC)** | ***M Age* (SD) of MCI and HC** | **Test Type and Cognitive Domain** | **Administration Mode** | **Reference Standard (Diagnosis and Criteria)** | **Sensitivity & Specificity** |
| --- | --- | --- | --- | --- | --- | --- |
| Alegret (2020, Spain) *FACEmemory* | 276 (122, 154) | 67.74 (7.93); 67.98 (7.92) | Traditional: Single-Domain (Memory) | Laboratory, Instruction-Based | MCI: Petersen Criteria (2004), Neuropsychological Battery | 0.74; 0.72 |
| Ambrosini (2024, Italy) *IA Voce* | 89 (44, 45) | 82.8 (4.6); 76.5 (4.9) | Traditional: Single-Domain (Language) | Laboratory, Examiner-Based | MCI: Petersen Criteria (2004), MoCA | 0.77; 0.73 |
| Berron (2024, Germany) *RDMC* | 103 (25,78) | 69.2 (6.79); 68.2 (5.47) | Traditional: Single-Domain (Memory) | Remote | MCI: Petersen Criteria (2004), CDR | 0.81; 0.72 |
| Boz (2019, Turkey) *Virtual Super Market* | 89 (37, 52) | 70.41 (7.3); 67.56 (6.04) | Novel: Multi-Domain (Memory, Executive Function) | Laboratory, Training-Based | MCI: Neuropsychological Battery | 0.73; 0.85 |
| Cabinio (2020, Italy) *Smart Aging Serious Game* | 139 (32, 107) | 76.47 (3.03); 76.75 (5.31) | Novel: Multi-Domain (Memory, Attention, Problem-Solving) | Laboratory, Examiner-Based | MCI: MoCA | 0.84; 0.76 |
| Caffò (2012, Italy) *VReoT* | 104 (51, 53) | 69.89 (5.17); 68.06 (5.96) | Novel: Multi-Domain (Attention, Executive Function) | Laboratory, Training-Based | MCI: MMSE | 0.80; 0.94 |
| Chan (2020, China) *Electronic Cognitive Screening* | 180 (54, 126) | 76.1 (6.3); 70.2 (8.1) | Traditional: Multi-Domain (Memory, Language) | Laboratory, Instruction-Based | MCI: MoCA | 0.83; 0.83 |
| Cheah (2022, Malaysia) *Digital Screening System* | 118 (59, 59) | 62.58 (5.89); 67.51 (6.30) | Traditional: Multi-Domain (Visuospatial Function, Memory) | Laboratory, Examiner-Based | MCI: MMSE | 0.85; 0.90 |
| Curiel (2016, USA) *MITSI-L* | 98 (34, 64) | 77.6 (6.3); 74 (7.3) | Traditional: Single-Domain (Memory) | Laboratory, Instruction-Based | MCI: Petersen Criteria (2004), MoCA | 0.85; 0.84 |
| Fristed (2022, Denmark) *Automatic Story Recall* | 151 (73, 78) | 69.58 (7.3); 70.37 (4.35) | Traditional: Single-Domain (Language) | Remote | MCI: CDR | 0.81; 0.73 |
| Fung (2020, Hong Kong) *HK-VMT* | 606 (97, 509) | 73.04(7.0); 69.5 (6.6) | Traditional: Multi-Domain (Memory, Attention) | Remote, Training-Based | MCI: MoCA | 0.86; 0.75 |
| Ichii (2019, Japan) *CogEvo* | 224 (103, 121) | 80.52 (6.1); 77.4 (7.2) | Novel: Multi-Domain (Executive Function, Memory) | Laboratory, Instruction-Based | MCI: CDR | 0.78; 0.54 |
| Klil-Drori (2024, Canada) *XpressO* | 88 (46, 42) | 70.34 (7.23) | Traditional: Multi-Domain (Language, Memory) | Remote | MCI: Petersen Criteria (2004), MoCA | 0.91; 0.90 |
| Konstantinidis (2021, Greece) *FitForAll (FFA)* | 102 (64, 38) | 69.3 (6.4); 67.1 (5.2) | Novel: Multi-Domain (Executive Function, Attention) | Laboratory, Training-Based | MCI: MMSE | 0.84; 0.63 |
| McMurray (2024, USA) *Brainfx SCREEN* | 147 (71, 76) | N/A | Novel: Multi-Domain (Memory, Executive Function) | Laboratory, Instruction-Based | MCI: MoCA | 0.63; 0.74 |
| Memoria (2014, Cuba) *CANS-MCI* | 76 (35, 41) | 73.80 (5.5); 71.68 (4.5) | Traditional: Multi-Domain (Memory, Attention) | Laboratory, Training-Based | MCI: Neuropsychological Battery | 0.80; 0.73 |
| Muller (2017, Germany) *dCDT* | 50 (30, 20) | 65.3 (6.6); 66.9 (9.4) | Traditional: Single-Domain (Visuospatial Function) | Laboratory, Instruction-Based | MCI: MoCA | 0.80; 0.70 |
| Oliva (2022, Spain) *NAIHA NeuroCognitive Test* | 114 (44, 70) | N/A | Novel: Multi-Domain (Memory, Attention) | Laboratory, Instruction-Based | MCI: MMSE | 0.84; 0.50 |
| Paterson (2022, UK) *BHA* | 91 (51, 40) | 75 (5.7); 74 (7.0) | Traditional: Multi-Domain (Memory, Attention) | Laboratory, Instruction-Based | MCI: MoCA | 0.57; 0.83 |
| Possin (2018, USA) *BHA* | 208 (71, 137) | 73.7 (9.9); 75.6 (6.3) | Traditional: Multi-Domain (Memory, Language) | Laboratory, Instruction-Based | MCI: MoCA | 0.85; 0.84 |
| Robens (2019, UK) *dTDT* | 131 (64, 67) | 67.9 (11.2); 65.9 (10.3) | Traditional: Single-Domain (Visuospatial Function) | Laboratory, Examiner-Based | MCI: MMSE | 0.56; 0.84 |
| Rodriguez-Salgado (2021, Cuba) *BHA Cuban version* | 99 (46, 53) | 72.7 (7.5); 70.4 (5.9) | Traditional: Multi-Domain (Memory, Language) | Laboratory, Instruction-Based | MCI: MoCA | 0.87; 0.85 |
| Wright (2011, USA) *DETECT* | 284 (112, 172) | N/A | Traditional: Multi-Domain (Memory, Attention) | Laboratory, Training-Based | MCI: MMSE | 0.84; 0.90 |
| Wu (2017, China) *E-CT* | 241 (129, 112) | 76.5 (7.49); 74.7 (6.94) | Traditional: Single-Domain (Visuospatial Function) | Laboratory, Examiner-Based | MCI: MMSE | 0.70; 0.79 |
| Yan (2021, China) *Virtual Super Market Program* | 126 (62, 64) | 82.00 (15.50); 73.5 (16) | Novel: Multi-Domain (Memory, Attention) | Laboratory, Training-Based | MCI: MoCA | 0.85; 0.73 |
| Ye (2022, USA) *BrainCheck Battery* | 57 (22, 35) | 73.5 (5.9); 67.8 (9.6) | Novel: Multi-Domain (Memory, Executive Function) | Remote | MCI: CDR | 0.86; 0.83 |
| Yip (2017, Taiwan) *CB-STM* | 52 (22, 30) | N/A | Traditional: Single-Domain (Memory) | Laboratory, Instruction-Based | MCI: MoCA | 0.76; 0.73 |
| Yu et al. (2015, USA) *MOCA-CC* | 118 (63, 55) | 73.6 (5.1); 72.2 (5.0) | Traditional: Multi-Domain (Memory, Executive Function, Attention) | Laboratory, Examiner-Based | MCI: MoCA, CDR, Clinical Diagnosis | 0.96; 0.87 |
| Zhang et al. (2023, China) *Hybrid TMT* | 198 (98, 100) | 72.47 (7.45); 69.83 (6.92) | Traditional: Single-Domain (Visuospatial Function) | Laboratory, Instruction-Based | MCI: MMSE | 0.77; 0.78 |
| Zhang et al. (2024, China) *Digital Geriatric Complex Figure* | 64 (38, 26) | 67.5 (7.2); 64.6 (7) | Traditional: Multi-Domain (Memory, Executive Function, Attention) | Laboratory, Instruction-Based | MCI: Clinical Diagnosis, Neuropsychological Battery | 0.79; 0.85 |
| Zhao et al. (2024, China) *DCS* | 308 (99, 209) | 74.1 (8.8); 69.8 (7.3) | Novel: Multi-Domain (Memory, Executive Function) | Laboratory, Training-Based | MCI: MoCA, CDR, Clinical Diagnosis | 0.90; 0.92 |
| Zygouris et al. (2015, Greece) *Virtual Super Market (VSM)* | 55 (34, 21) | 70.32 (1.22); 66.57 (1.2) | Novel: Multi-Domain (Memory, Executive Function) | Laboratory, Training-Based | MCI: Neuropsychological Battery | 0.85; 0.81 |

**Figure S1. Funnel plots for sensitivity and specificity.**

Funnel plots displaying the distribution of included studies in the meta-analysis for (A) sensitivity and (B) specificity. Each point represents individual study. The symmetry of the plots is used to assess potential publication bias or small-study effects. The x-axis shows the reported Logit of sensitivity or specificity, while the y-axis represents the standard error (SE).

**Stata** **Script** used to assess Diagnostic accuracy of meta-analysis, forest plots, meta-regression, and funnel plots to assess heterogeneity and publication bias.

Stata Version 18

1. Load data

import delimited "diagnostic_mci_data.csv", clear

2. Bivariate HSROC model (Rutter & Gatsonis)

metandi tp fn fp tn, or

Optional: visualization

metandiplot

3. Forest plots of sensitivity and specificity (descriptive)

gen sens = tp / (tp + fn)

gen spec = tn / (tn + fp)

metan sens spec, lcols(study_id)

4. Extract logit-transformed sensitivity and specificity

gen logit_sens = log(sens / (1 - sens))

gen logit_spec = log(spec / (1 - spec))

5. Compute variances

gen var_logit_sens = 1/tp + 1/fn

gen var_logit_spec = 1/tn + 1/fp

6. Bivariate HSROC model (Rutter & Gatsonis)

metadta tp tn fp fn, studyid(study_id)

6a. Extract and store heterogeneity parameters

matrix list e(b)

matrix list e(V)

7. Meta-regression on logit-sensitivity

metareg logit_sens quadas_rob1 quadas_rob2 quadas_rob3 quadas_rob4*** ac1 ac2 ac3 mean_age tool_type mode_admin, wsse(var_logit_sens)

8. Meta-regression on logit-specificity

metareg logit_spec quadas_rob1 quadas_rob2 quadas_rob3 quadas_rob4*** ac1 ac2 ac3 mean_age tool_type mode_admin, wsse(var_logit_spec)

9. Funnel plot for publication bias (descriptive)

metafunnel logit_sens var_logit_sens, title("Funnel plot: logit Sensitivity")

metafunnel logit_spec var_logit_spec, title("Funnel plot: logit Specificity")
